# Supplementary material for: Associations of Social and Psychological Resources with Different Facets of Chronic Stress: A Study with Employed and Unemployed Adolescents
Source: Int J Environ Res Public Health. 2020 Jul 13;17(14):5032. doi: 10.3390/ijerph17145032 (PMC7400050; doi:10.3390/ijerph17145032)
Supplement: Supplementary file 1 [file ijerph-17-05032-s001.pdf]

Supplementary Materials:

Table S1. Pearson correlation matrix for continuous variables (N = 1405).

	1	2	3	4	5	6	7	8	9	10	11	12
1. SCR	1											
2. WRY	0.87 **	1										
3. OVRL	0.78 **	0.62 **	1									
4. SOVRL	0.66 **	0.56 **	0.61 **	1								
5. DIS	0.66 **	0.56 **	0.57 **	0.53 **	1							
6. RECO	0.70 **	0.48 **	0.58 **	0.55 **	0.63 **	1						
7. ISO	0.69 **	0.62 **	0.57 **	0.51 **	0.58 **	0.54 **	1					
8. SocS	−0.49 **	−0.45 **	−0.40 **	−0.36 **	−0.40 **	−0.40 **	−0.61 **	1				
9. FamS	−0.38 **	−0.35 **	−0.32 **	−0.33 **	−0.30 **	−0.31 **	−0.35 **	0.37 **	1			
10. GSE	−0.28 **	−0.29 **	−0.19 **	−0.08 **	−0.21 **	−0.16 **	−0.25 **	0.17 **	0.16 **	1		
11. SelfE	−0.54 **	−0.56 **	−0.38 **	−0.32 **	−0.38 **	−0.32 **	−0.46 **	0.34 **	0.32 **	0.38 **	1	
12. Age	0.05	0.05 *	0.06 *	0.08 **	0.08 **	0.05 *	0.06 *	−0.12 **	−0.04	−0.004	−0.05	1

SCR = Chronic stress screening scale, WRY = stress due to chronic worrying, OVRL = stress due to work overload, SOVRL = stress due to social overload, DIS = stress due to occupational discontent, RECO = stress due to a lack of social recognition, ISO = stress due to social isolation, WRY = stress due to chronic worrying, SocS = satisfaction with social support, FamS = family support, GSE = self-efficacy, SelfE = self-esteem.* $p < 0.05$, ** $p < 0.001$.
